# Electrospun Composite Nanofibers for Functional Applications

**DOI:** 10.3390/polym14112290

**Published:** 2022-06-05

**Authors:** Sana Ullah, Motahira Hashmi, Ick Soo Kim

**Affiliations:** 1 Graduate School of Medicine Science and Technology, Department of Science and Technology, Division of Smart Material, Shinshu University, Tokida 3-15-1, Ueda, Nagano 386-8567, Japan; 2 Nano Fusion Technology Research Group, Institute for Fiber Engineering (IFES), Interdisciplinary Cluster for Cutting Edge Research (ICCER), Shinshu University, Tokida 3-15-1, Ueda, Nagano 386-8567, Japan

Summary of the Special Issue:

Nanotechnology has deep roots in solving advanced and complex problems ranging from life sciences to water purification, from energy to structural applications, and from sensors to sustainable development. This Special Issue focuses on some of the applications of electrospun nanofibers in these areas. Our research group is working on applications of electrospun nanofibers in air filtration and facemasks [1,2], wound dressings [3,4,5,6,7,8,9,10,11,12,13,14,15,16,17,18,19,20], sensors [14], water purification [21], blood vessels [22], axons [23], adsorption [24,25], photosensitive materials [26], electronics [27,28], and various other fields of science and technology. 

An interesting study was conducted on the fabrication of poly(ethylene-glycol 1,4-cyclohexane dimethylene-isosorbide-terephthalate) electrospun nanofiber mats (PEICT ENMs) for the potential infiltration of fibroblast cells. In this study, morphological, structural, and cytotoxicity assessments were performed. It was found that the PEICT nanofibers possessed excellent biocompatibility. Hence, the results confirmed that PEICT ENMs can potentially be utilized as a biomaterial [29]. Silver nanoclusters are also considered excellent antibacterial agents; another study in this Special Issue synthesized novel sericin-encapsulated silver nanoclusters (sericin-AgNCs) through a green synthesis route. Subsequently, these sericin-AgNCs were incorporated into ultrafine electrospun cellulose acetate (CA) fibers to assessing their antibacterial performance. It was confirmed via antibacterial activity testing that the AgNCs were sufficiently antibacterial in combination with cellulose acetate nanofibers to allow this nanofibrous web to be used as an antibacterial wound dressing or in similar applications. It was also observed that the addition of 0.17 mg/mL sericin-AgNCs to electrospun cellulose acetate fibers showed antibacterial activity of around 90%, while this value was increased to 99.9% by increasing the Ag nanocluster amount to 1.17 g/mL (i.e., increasing by 10 times) [30]. A comparison was drawn between polybutylene succinate (PBS) membranes and electrospun polyvinylidene fluoride (PVDF). Polymer fibers fabricated by rolling electrospinning (RE) and non-rolling electrospinning (NRE) showed different mechanical and morphological properties. Graphene oxide (GO) composite is neutral with regard to effects on mechanical properties. The PBS membrane offers a higher pore area than electrospun PVDF and can be used as a filter. The protein capture efficiency and protein staining were analyzed via the SPMA technique using albumin solution filtration. The fabricated membranes were compared with commercially available filters. RE with GO and PBS showed two times greater capture capacity than commercial membranes and more than sixfold protein binding as compared to the non-composite polymer. Protein staining results further verified the effectiveness of the fabricated membranes by showing a darker stain color [31]. In another research area, an in vitro cyst model was prepared in which hollow nanofiber spheres were developed, named “nanofiber-mâché balls.” Electrospun nanofibers of a hollow shape were fabricated on alginate hydrogel beads. A fibrous geometry was provided by the balls having an inner volume of 230 mm^3^. A route for nutrients and waste was developed by including two ducts. This oriented migration was produced with a concentration gradient in which seeded cells attached to the inner surface, resulting in a well-oriented structure. Fibers attached to the internal surface between the duct and hollow ball restrict cell movement and do not allow them to exit the structure. An adepithelial layer on the inner surface was developed with this structure, and the nanofiber-mâché technique is most suitable for investigating cyst physiology [32]. The affinity of electrospun polyvinyl alcohol (PVA) nanofiber fabric was improved by modification with Cibacron Blue F3GA (CB). The bovine hemoglobin (BHb) adsorption capacity of the nanofiber fabrics at different concentrations of protein before and after treatment was studied in batch experiments. The original fibers possessed a BHb adsorption capacity of 58 mg/g, while the modified nanofiber fabric showed a capacity of 686 mg/g. The feed concentration and permeation rate were investigated. Static adsorption was performed to investigate the impact of the pH on BHb and bovine serum albumin (BSA) adsorption. Selective separation experiments were carried out at the optimal pH value. A reusability test was carried out by performing three adsorption–elution cycles [33]. Task-specific functionalized polymeric nanofiber mats were fabricated from homocysteine thiolactone and polyvinylpyrrolidone through electrospinning. This allows for post functionalization by a thiol–alkene “click” reaction. Modifications to electrospun nanofibers were made by introducing different functionalities under controlled conditions without damaging the nanostructure of the nanofibers. The results showed that modified nanofibers can be used for sensing and catalytic applications [34]. Recent trends in research showed that a large surface area, pore size, and geometry of nanofibers make them suitable for filtration applications. The filtration efficiency can be modified through nanonets of a smaller diameter than nanofibers. Polyacrylonitrile (PAN) was used to form nanonets, and their filtration efficiency was analyzed. Electrospun polyacrylonitrile acetyl methyl ammonium bromide (PAN–CTAB) possessed improved mechanical and thermal properties. PAN–CTAB nanofiber/nanonets showed 99% filtration efficiency as air filters and a low pressure drop of 7.7 mm H_2_O at an air flow rate of 80 L/min. This study provides a new approach to the fabrication of air filters with higher filtration efficiency [35]. Another milestone in the field of skin care was achieved with the development of effective hydrophilic nanofibers (NFs) loaded with folic acid (FA). Electrospinning and electrospraying techniques were used. The morphological, thermal, mechanical, chemical, in vitro, and cytocompatibility properties were analyzed, and the results showed that the fabricated nanofiber mat has the potential for use as wound dressings and in tissue engineering applications [19]. A study on solid-state batteries (SSBs) gained attention for its efficiency in energy density and high-safety energy storage devices. Researchers have made various efforts to fill the gaps for thin solid-state-electrolytes (SSEs) with regard to their ionic conductivity, thermal stability, and mechanical strength. Composite polymer electrolyte (CPE)-reinforced PI nanofiber with succinonitrile-based solid composite electrolytes were developed to fill this gap. CPE showed a high ionic conductivity of 2.64 × 10^−4^ S cm^−1^ at room temperature. The developed material is fire resistant, is mechanically strong, and offers promising safety [36]. Research goals of controllable release and antibacterial properties were achieved via orange essential oil (OEO) and silver nanoparticles (AgNPs) deposited on a cellulose (CL) nanofiber mat. The fabrication of the finished nanofiber mats involved different steps like deacetylation and coating of silver nanoparticles prepared in OEO solution by an in situ method with two different concentrations. The successful deposition of AgNPs incorporated in the OEO was analyzed via SEM-EDS, TEM, XRD, and FT-IR. The tensile strength was recorded after each step of the treatment and compared with that of CA nanofibers. Well-treated nanofiber mats showed good antibacterial activity against Gram-positive and Gram-negative bacteria [37]. Researchers are keen to find high-quality nanomaterials for medical applications to change the future of medicine. The similarity between human tissues and electrospun nanofibers has opened new doors for researchers in the field of the medical applications of nanofibers. Until now, electrospun nanofibers have been restricted to tissue scaffolding applications, but a combination of nanoparticles with nanofibers could provide better function in photothermal, magnetic response, biosensing, antibacterial, and drug delivery applications. There are two methods to prepare hybrid nanofibers and nanoparticles (NNHs): electrospinning is the easy and simple way, and the other way is self-assembly. Both methods have been adopted to achieve drug release, antibacterial, and tissue engineering applications [38].
